# Telehealth in community pharmacy: A new “place” for the appointment-based model given COVID-19 and the future of health care

**DOI:** 10.1177/17151635211014922

**Published:** 2021-05-24

**Authors:** Sara Rezahi, Annalise Mathers, Pooja Patel, Tiana Tilli, Lisa Dolovich

**Affiliations:** Leslie L. Dan Faculty of Pharmacy, University of Toronto, Toronto; School of Pharmacy, University of Waterloo, Waterloo; Leslie L. Dan Faculty of Pharmacy, University of Toronto, Toronto; Leslie L. Dan Faculty of Pharmacy, University of Toronto, Toronto; Whole Health Pharmacy Partners, Markham, Ontario; Leslie L. Dan Faculty of Pharmacy, University of Toronto, Toronto

Coronavirus disease 2019 (COVID-19) is a global pandemic, with over 133 million cases and nearly 3 million deaths confirmed worldwide as of April 2021.^1^ As the virus is transmitted via droplet contact, global recommendations suggest that individuals practise physical distancing and avoid nonessential activities outside their home to prevent community-based transmission.^2,3^ Vulnerable populations, such as those over 60 years of age, those with chronic illnesses or individuals with multimorbidities who are on multiple and/or complex medication regimens, are at increased risk of contracting COVID-19 and experiencing subsequent complications and poor outcomes.^4,5^ While pharmacies remain open as essential services, many people using pharmacy services belong to these vulnerable populations and so are less likely to visit their pharmacy.^5^ The pandemic affects continuity of health care and medication management, as patients may be uncomfortable or unable to leave their homes due to risk of exposure.

Telehealth is defined as a service that effectively connects individuals and their health care providers when an in-person interaction is not clinically necessary and facilitates health care provider-to-patient consultation.^6^ The most commonly used telehealth approaches are virtual visits, chat-based interactions, remote patient monitoring and technology-enabled modalities used for patient education or interprofessional communication.^6^ Virtual visits are synchronous, interactive encounters between a patient and a health care provider via video, telephone or live chat.^6^ Although virtual services within primary health care settings were available prior to the COVID-19 pandemic and many patients expressed interest in using these services, uptake of virtual care was slow to emerge in primary care until the pandemic; since this time, there has been a significant rise in the use of virtual care services.^7^

In Canada, it is estimated that two-thirds of primary care physician patient visits are now virtual, in comparison to an estimated 4% prior to the pandemic.^8^ Furthermore, a retrospective review of primary care physicians and patients who used virtual care services for 18 months showed that virtual visits could replace in-person visits and that 51% of patients had completed at least 1 virtual visit.^9^ Research is now also beginning to emerge that presents guidelines for optimizing the use of virtual care for specific chronic conditions such as diabetes.^10^ The COVID-19 pandemic provides a tremendous opportunity to expand the use of telehealth in pharmacy to reach people in their homes and ensure continuing delivery of care.

Virtual care, including telephone or video visits, has been part of the delivery of pharmacy services for many years. Systematic reviews show that pharmacy clinical services delivered by telephone have a positive impact on medication adherence, patient self-management of chronic disease and clinical disease outcomes such as controlled blood pressure and blood sugar in hypertension and diabetes, respectively.^11-13^ In a study comparing the effect of home blood pressure telemonitoring (biweekly phone calls by the pharmacist that taper to every 2 months) combined with pharmacist management vs usual care, the number of patients with controlled blood pressure was significantly higher in the pharmacist telemonitoring group, with this group showing increased medication adherence.^14^ Another study demonstrated that patients receiving pharmacist telemonitoring had a significant reduction in their A1c compared to a retrospective chart review of characteristically similar patients who received usual care (2.07% vs 0.66% decrease in A1c).^15^ Virtual care provision in rural areas has effectively identified medication discrepancies and drug therapy problems through video chat.^16^ The COVID-19 pandemic has accelerated the need for pharmacy to integrate virtual approaches into routine medication management services and programs to promote virtual continuity of care and improve patient health.

The appointment-based model (ABM) is an example of a proactive approach to pharmacy practice that synchronizes chronic prescription refills and couples it with regularly scheduled patient-pharmacist appointments to optimize medication management.^17-21^ The ABM is a common approach in the United States and is gaining traction in Canada.^22^ Medication synchronization involves aligning chronic medications to 1 refill date so that all medications can be picked up on 1 scheduled day. While the synchronized pickup date can vary, most ABM models in the literature use a 30-day interval, with some using a 90-day interval.^17-21^

The general steps of the ABM are shown in Table 1.^23,24^ Historically, the ABM interaction was conducted entirely in person and required the patient to be physically present for the pharmacist appointment and medication pickup. The ABM approach has enormous potential to transition to virtual care.

**Table 1:** Implementing the appointment-based model

Getting your pharmacy set up	• Set goals • Educate your pharmacy team • Select a staff member to lead the program each shift • Create a mobile area to make calls and complete documentation • Organize all necessary paperwork
Patient identification and enrolment	• Identify patients who would benefit from the ABM program • Explain the ABM and inform patient of its benefits when patient brings new or refill prescriptions to the pharmacy • Obtain patient consent
Completion of enrolment	• Update pharmacy management system to indicate patient is enrolled • Add the patient appointment to an online pharmacy calendar • Follow up with your patient’s primary care physician
Medication synchronization	• Discuss with the patient which medications will be synchronized • Decide on a cycle length with the patient (7, 14, 28, 56 or 84 days) • Align the medications using short and long fills
5 days before the appointment	• Call all patients who have a scheduled appointment in 5 days to confirm prescriptions to be filled and identify changes, if any • Print the patient’s medication profile to use as a guide for the conversation. Make notes, scan them into the patient’s profile, then shred them. • Fill all the synced and PRN medications that the patient requested. This allows enough time to resolve any prior authorization, refill and inventory issues. Bag any OTC/vitamins the patient needs and charge them at the end of the appointment.
1 day before the appointment	• The pharmacist should look over the notes from the phone call a few days prior. • Determine what issues to address and how long the appointment will take. • The lead of the day should make sure that all requested medications were filled. Call and confirm the time, date and length of appointment with the patient. If the patient is unable to make the appointment, reschedule the appointment.
The appointment	• Address any patient issues/concerns. • Document and follow up with the primary care physician, as needed. • The pharmacist engages in clinical services (e.g., medication review, deprescribing, immunizing, monitoring, counselling). • Schedule the next appointment.
Daily maintenance	• Check the prescription pickup bin and reschedule missed appointments. • Ensure all patient forms have been scanned in and shredded.

OTC, over-the-counter; PRN, pro re nata (as needed).

The ABM has multiple benefits for both patients and pharmacists. Scheduled appointments provide an opportunity to plan for how to address medication-related issues (e.g., drug changes, side effect management) and optimize medication regimens (e.g., perform medication reviews, deprescribing). It provides pharmacists with an opportunity to fulfill their role as health promoters to plan ahead and offer, where relevant, adult immunizations, lifestyle modification education, disease state monitoring and referrals to other health care professionals. The ABM may also reduce medication nonadherence,^25^ since scheduled appointments to pick up medications decrease the number of trips to the pharmacy and increase number of refills.^18-21^ Having synchronized and scheduled refill dates creates a predictable workflow and increases pharmacy revenue and so the ABM offers increased, scheduled opportunities for pharmacists to conduct billable services and potentially increase pharmacy revenue.^26,27^

Recognizing the effectiveness of ABM, we considered the challenge of providing ABM in a model that incorporates telehealth approaches, given the new obstacles presented by COVID-19. Key findings from the literature guided decision-making on how to adapt a conventional ABM model:

• A previous ABM study comparing in-person only, in-person initial meeting with telephone follow-up and telephone-only care all improved adherence and increased prescription refill numbers.^17^
• Research focusing on telephone-based interventions for the ABM has shown improvement in medication adherence.^18-21^
• While there is no evidence for video-based ABMs within pharmacy, literature comparing video and telephone physician consultations shows that both video and telephone consults are of similar length and quality, but video has more technical difficulties.^28^
• Studies of older adults over 55 years of age suggest a preference for telephone rather than video consultations due to barriers including audiovisual difficulties (e.g., difficulty hearing or reading onscreen text), reduced cognitive ability (e.g., remembering passwords), Internet connection issues and low computer literacy (e.g., inexperience in computer use).^29,30^
• Telephone-based virtual models not only align with patient preference but are also more accessible in Canada. According to Statistics Canada, 99% of adults aged 65 and above in Ontario have telephone access, while only 80% have Internet access.^31,32^ Only 30% have used the Internet for video streaming or calling.^33^

Given the current evidence, our recommendations for adaption of a conventional in-person ABM can be seen in Table 2.

**Table 2:** Implementing the appointment-based model: A comparison of modified steps

Steps modified	Conventional ABM	Virtual ABM
Work area and staff member	• Select a staff member to lead the program each shift. • Create a mobile area to make calls and complete documentation. • Organize all necessary paperwork within the patient’s pharmacy profile.	• See conventional ABM. • Employ a telephone-based ABM model as the main mode of interactions. Offer video consultations only if platform is secure and ensure that the Internet connections are sufficient and the patient is comfortable with technology.
Patient identification and enrolment	• Identify patients who would benefit from the ABM program. • Update patient medication profile if necessary. • Explain the ABM and inform patient of its benefits when patient brings new or refill prescriptions to the pharmacy. • Obtain patient consent.	• See conventional ABM. • Conduct an intake meeting via telephone to perform initial medication synchronization and schedule future appointment frequency.
Before the appointment	• Call all patients who have a scheduled appointment in 5 days to confirm prescriptions to be filled and identify changes, if any. • Print the patient’s medication profile to use as a guide for the conversation. Make notes, scan them into the patient’s profile, then shred them. • Fill all the synced and PRN medications that the patient requested. This allows enough time to resolve any prior authorization, refill and inventory issues. Bag any OTC/vitamins the patient needs and charge them at the end of the appointment.	• Schedule a preappointment telephone call 7 days prior to the pharmacist-patient appointment to confirm refills and appointment. This was increased from 5 days to allow for more time to implement medication changes, order medication or reach out to the prescriber if needed. • Arrange a 1-day prior reminder call. • These calls can be automated at the discretion of the pharmacy. Automated reminders have been shown to improve refill rates but can be expensive.^34-36^
The appointment	• Address any patient issues/concerns. • Document and follow up with the primary care physician as needed. • The pharmacist engages in clinical services. • Schedule the next appointment. • Patient picks up their synchronized medication refills.	• See conventional ABM. • A regularly scheduled telephone appointment, scheduled in close temporal proximity to the medication delivery, enables patients and pharmacists to proactively address medication and health-related issues. • Synchronized medication delivery to patients reduces in-person visits and potential exposure. • During COVID-19, Canadian pharmacies have seen a doubling in the volumes of medication deliveries.^37^ • Medication synchronization can reduce delivery expenses by reducing the number of unique deliveries required per patient.

ABM, appointment-based model; OTC, over-the-counter; PRN, pro re nata (as needed).

Implementing ABM to meet the needs of high-risk patients amid the COVID-19 pandemic has the potential to serve as a precedent for future virtual ABMs well beyond the COVID-19 pandemic for patients who are unable to visit a pharmacy in person due to physical or health-related barriers. Furthermore, providing medication synchronization, preappointment calls and appointments via telephone, coupled with medication delivery, can allow for increased access to pharmacy services and will ensure patients continue to have access to pharmacy services as the COVID-19 pandemic continues. Health care is shifting to a virtual model, and it is imperative that pharmacy shifts along with it. ■
